# Limitations of VCG based gating methods in ultra high field cardiac MRI

**DOI:** 10.1186/1532-429X-15-S1-W19

**Published:** 2013-01-30

**Authors:** JW Krug, G Rose, D Stucht, G Clifford, J Oster

**Affiliations:** 1 Chair for Healthcare Telematics and Medical Engineering, Otto-von-Guericke University of Magdeburg, Magdeburg, Germany; 2 Department of Biomedical Magnetic Resonance, Otto-von-Guericke University of Magdeburg, Magdeburg, Germany; 3 Department of Engineering Science, University of Oxford, Oxford, UK

## Background

The electrocardiogram (ECG) is important for gating purposes in cardiac magnetic resonance imaging (CMR). However, the magnetohydrodynamic (MHD) effect, which is caused by the flow of blood in the static magnetic field, makes it difficult to record clean ECG signals for gating. The vectorcardiogram (VCG), which can be derived from the ECG signal, is commonly used for gating purposes and has been well established for magnetic fields strength of up to 3T. However, for higher field strengths this method is prone to errors [1]. This work tries to explain the reasons for the VCG based methods not being suitable for cardiac gating in ultra high field MRI.

## Methods

ECGs were recorded using a standard 12-lead Holter ECG (Getemed, Germany) inside a 7T MR scanner (Siemens Magnetom), a 3T MR scanner (Philips Achieva) and outside the MR scanner as references. Measurements were made on five healthy volunteers while MR imaging was switched off. The VCGs were derived from the 12-lead ECG using the inverse Dower matrix [2]. The VCG method [3] was implemented and applied to the acquired data sets. This method measures the distance in the VCG space between a static reference vector defined by the R-wave (R) peak of an ECG signal recorded outside the MR scanner (at 0T) and the VCG vector recorded inside the MR scanner. Those points where a minimal distance and angle between both vectors is reached are classified as R peaks.

## Results

**Figure 1:**
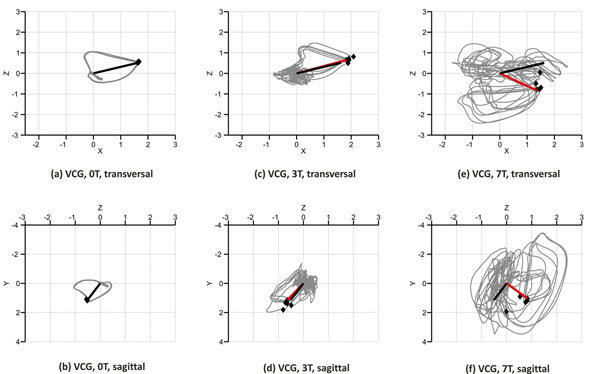
Spatial representation of the VCGs recorded at 0T, 3T and 7T. The black vector is used as the reference vector obtained from the ECG recorded at 0T. The red vector corresponds to one R peak from the 3T and the 7T measurement, respectively. Black dots mark the actual, manually annotated positions of the R peaks.

**Figure 2:**
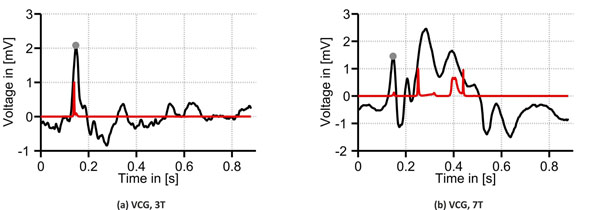
VCG lead x (black) from the 3T and 7T measurements. The red graphs correspond to the minimum distance between the static reference vector and the actual VCG signal [3]. The red graph shows several peaks for the 7T measurement. Gray dots mark the position of the R peak.

A spatial representation of the VCGs recorded at 0T, 3T and 7T is given in Figs. 1(a)-(f). When comparing the VCGs recorded at 0T and 3T, the mean angle between the static reference and the R peaks at 3T was 3.6° whereas the mean difference between the norms of these vectors was 0.4mV. For the 7T measurements, the mean angle and the mean difference between the norms of the static reference and the R peak vectors were 68.7° and 1.7mV, respectively. As depicted in Fig. 2(b), the minimum distance algorithm [3] showed several minima for the ECG signal recorded in the 7T scanner which was not the case for the 3T measurements (Fig. 2(a)).

## Conclusions

The angle of the R peak vector substantially changed for the 7T measurement. This can be explained by the fact that the MHD effect has an intrinsic DC component. Since this DC component was removed by the ECG recorder’s high pass filter, the amplitude of the R peaks in each axis of the VCG signal changed which yielded an alteration of the R peak's angle. Additionally, the 7T VCG space is severely corrupted by the strength and complexity of the MHD signal.

## Funding

This work was founded by the Federal Ministry of Education (Germany, BMBF, 03IP710) and by the Royal Academy of Engineering (UK).
